# Genotoxic and cytotoxic action potential of Terminalia citrina, a medicinal plant of ethnopharmacological significance

**DOI:** 10.17179/excli2016-551

**Published:** 2016-10-24

**Authors:** Muhammad Furqan Akhtar, Ammara Saleem, Ali Sharif, Bushra Akhtar, Maaz Bin Nasim, Sohaib Peerzada, Moosa Raza, Hira Ijaz, Shoaib Ahmed, Maryam Shabbir, Sajid Ali, Zeeshan Akbar, Syed Saeed Ul Hassan

**Affiliations:** 1 Faculty of Pharmacy, The University of Lahore, Lahore, Pakistan; 2 Faculty of Pharmaceutical Sciences, GC University, Faisalabad, Pakistan; 3 Institute of Pharmacy, Physiology and Pharmacology, University of Agriculture, Faisalabad, Pakistan; 4 Faculty of Pharmacy, University of Sargodha, Sargodha, Pakistan; 5 Institute für pharmazeutische Technologie & Biopharmazie, Philipps University Marburg, Germany

**Keywords:** genotoxicity, mutagenicity, herbs, cytotoxicity, alkaloids, ethnomedicine

## Abstract

Most herbal medicines utilized in complementary and alternative medicine lack safety evaluation setting our lives under unwarranted risks. Present study comprised of genotoxic and cytotoxic appraisal of *Terminalia citrina* fruits which are used as a folklore medicine for treatment of various ailments. Aqueous and ethanolic extracts of *T. citrina* fruit extracts were evaluated for the presence of different phytochemicals. Genotoxic potential of both the extract of *T. citrina *was assessed through Ames reverse mutagenicity assay in Salmonella TA 100 and 102 strains. Cytotoxic potential of *T. citrina* was determined in baby hamster kidney cell line (BHK-21). Statistical analysis was carried out by ANOVA following post hoc test. Phytochemical analysis showed the presence of alkaloids, carbohydrates, phenolic compounds, tannins, catechins and saponins. It was revealed that both the extracts of *T. citrina* exhibited significant mutagenicity in tester strains. Ethanolic extract showed higher mutagenicity in TA 100 strain, whereas aqueous extract of *T. citrina* exhibited higher mutagenicity in TA 102 strain than TA 100. Both the extracts of *T. citrina *showed dose-dependent mutagenicity. Fifty percent cell viability was exhibited by 260 and 545 µg/mL of ethanolic and aqueous extracts respectively. This study concludes that the ethanolic and aqueous fruit extracts of *T. citrina *may not be safe owing to their mutagenic and cytotoxic potential and it necessitates further investigation regarding its safety evaluation.

## Introduction

A large population of developing countries utilizes plant or herb based formulations and preparations owing to their low cost and less severe adverse effects (Akhtar et al., 2016[3]). However, most herbs and their preparations may not be safe due to the presence of a large number of phytochemicals. A very few studies reporting the safe and effective use of medicinal plants and their preparations have been carried out (Abed et al., 2013[2]).

*T. citrina,* a deciduous tree, is of extensive ethno-medicinal significance in South Asia and Africa. It is widely distributed throughout the forests of Bangladesh and India. Different parts of this plant are used for the management of various diseases on folklore basis. The fruits of *T.* *citrina* are used in the management of chronic fever and loss of appetite. These are also used as sexual stimulant (Hossan et al., 2009[15]). Fruits of *T. citrina* have also been used for the treatment of diarrhea and other digestive disorders. These fruits have also been employed as anthelmintic in some regions of Middle East (Amiri and Joharchi, 2013[7]). These fruits are also used as an anti-inflammatory, analgesic and antipyretic remedy. Fruits of *T. citrina* are also used in the treatment of asthma, constipation, boils, migraine, dental disorders, dizziness, hemorrhoids, anemia, eye diseases, infections, traumatic cuts, cardiac disorders, hepatomegaly and urolithiasis (Soe, 2004[25]). Other parts of this plant have also some ethnomedicinal uses such as bark being used as a diuretic and cardiotonic while seeds are useful in gastritis and colitis (Khare, 2007[17]). 

*T. citrina *(Gaertn.) Roxb. is also known as Haleela Zard (Ingle and Dhabe, 2011[16]). It belongs to genus *Terminalia* and family Combrataceae with approximately 200 species. It is distributed throughout the moist, semi moist, tropical and sub-tropical areas of the world. Approximately, 24 species of this genus are found in different regions of South Asia (Srivastav, 2003[27]). Among these species, *T. arjuna, T. bellerica, T. chebula, T. tomentosa *and *T. catappa *exhibit extensive medicinal and economic significance. The taxonomic status of *Terminalia* is disputed due to the existence of its various morphological types. Some taxonomists have subdivided the genus into sections or subgenera on the basis of fruit characteristics (Srivastav et al., 2002[28]).

In-vitro toxicological assays have gained pronounced significance for the safety evaluation of drugs. Majority of the chemicals exhibiting mutagenic appraisal also demonstrate carcinogenic potential (Kristanc and Kreft, 2016[18]). Genotoxicity is an imperative characteristic of the toxicants which usually results from the mutations in hereditary characteristics or breakage of DNA strands. It leads to apoptosis, carcinogenesis or alteration of phenotype. Ames reverse mutation test is a reliable tool for the assessment of mutagens of synthetic, environmental or natural origin (Sponchiado et al., 2016[26]). This assay has gained widespread significance because of its ability to detect mutagens and its short term testing nature (Sharif et al., 2016[24]). 

Cytotoxicity can be considered a strong indicator of *in vitro *toxicity (Mohanty, 2014[20]). MTT assay, based upon tetrazolium dye, is a colorimetric method in nature. It is one of the cytotoxicity testing methods which demonstrate the *in vitro *cell viability. It serves as an indicator of the cell activation, cytotoxicity and anti-proliferative activity of drugs, toxicants and environmental pollutants (Lupu and Popescu, 2013[19]). MTT is converted into measureable formazan via the action of mitochondria, cell membrane and cytoplasm based oxido-reductases present in viable cells (Berridge et al., 2005[10]). 

This study comprised of the assessment of mutagenic potential and* in vitr*o cytotoxic action of the aqueous and ethanolic extracts of *T. citrina* fruits.

## Materials and Methods

All the chemicals were acquired from Sigma Aldrich^®^, BDH^®^ and thermo fisher Scientific^®^. Dried fruits of *T. citrina* obtained from the local market, were identified from GC University, Lahore. The sample was preserved in local herbarium and its voucher number was “GC. Herb. Bot. 2205”. The aqueous and ethanolic extracts were prepared with a soxhlet apparatus and evaporated with a rotary evaporator at 40 °C. Extracts were freeze dried and stored in a freezer at -20 °C until further use.

### Phytochemical evaluation of plant extracts

The ethanolic and aqueous extracts of *T. citrina *fruits were evaluated for the presence of alkaloids, amino acids, glycosides, polyphenols, tannins, catechins and saponins (Begum et al., 2015[9]). 

### Evaluation of genotoxicity

Ames mutation assay was carried out for the evaluation of mutagenic potential of *T. citrina* fruit extracts using two Salmonella Typhimurium TA 100 and TA 102 strains. These bacterial strains were procured from environmental bio-detection products Inc. Canada (bdpi). 

Salmonella strains were confirmed for their dependence on histidine and biotin and ampicillin resistance prior to genotoxicity testing (Mortelmans and Zeiger, 2000[21]). Sodium azide was used as a positive control for TA 100, whereas hydrogen peroxide served as positive control for TA 102. Distilled water was negative control. *T. citrina* fruit extracts were dissolved in distilled water with 150 mg/mL concentration. Extracts were diluted with distilled water with the tenfold dilution method. 1 mL extract or dilution and 0.1 mL of tester strain were mixed with vortex mixer. These extracts treated tester strains were incubated for one hour at 37 °C and mixed with low melting point agarose. Top layer of agar was prepared by mixing this melting point agarose with trace amounts of histidine and biotin. This top agar was poured on glucose minimal agar plates and incubated at 37 °C for 24 hrs (Akhtar et al., 2016[4]). Revertant colonies were counted manually. Mutagenic Index was calculated by following formula:


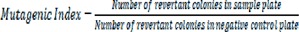


*T. citrina* extracts were considered as possible mutagen if mutagenic index was 2 or more, significantly mutagenic if mutagenic index was 3 or more and was considered highly mutagenic if mutagenic index was more than 4.

### Cytotoxicity assay

In cytotoxicity assay, baby hamster kidney cells (BHK-21) cell line was used. *In vitro* cytotoxicity assay of *T. citrina* was carried out on this cell line by means of frequently used Methyl-Thiazole Tetrazolium (MTT) assay. BHK-21 cells were obtained from Quality Operation Laboratory (QOL), UVAS, Lahore. Cell culture medium was prepared by dissolving 1.2 gm Dulbecco's minimum essential medium (DMEM) in 100 mL double distilled water. 10 % fetal bovine serum was added for growth medium and 1 % for maintenance medium. Cryopreserved BHK-21 cells were defrosted. Sample tubes containing BHK-21 cells were removed after decontamination with 70 % ethanol and thawed with an increase in temperature at a rate of 1 °C per minute. Stock of thawed cells was transferred to falcon tubes. The old medium was removed by centrifugation at 2000 rpm for 3 minutes (Akram et al., 2013[6]). 5 mL DMEM was used for washing and dilution of cell pellet. Cell counting was carried out with modified neubur's chamber. BHK-21 cells were seeded in a cell culture flask of 25 cm^2^ and placed in a carbon dioxide incubator at 37 °C. Cells were observed on daily basis for 90 % confluent cell layer. Viable cells were counted with 0.4 % trypan blue as it stained dead cells only.

Cell culture medium (DMEM) was used as positive control for assessment of cell viability whereas 20 % DMSO was used as negative control. Cells were propagated in 96 well-plates by using confluent monolayer bearing flasks. Cell culture media were removed from flasks. 5 mL trypsin (0.25 %) was added to detach the cells in each flask while incubating and mild shaking at 37 °C for 5 minutes. Growth media were used for washing and dilution, after that cell concentration was adjusted up to 10^5 ^cells/ml. Cell suspension (100 µL) was poured in each 96 well plate of 96 well plates, which were incubated at 37 °C in carbon dioxide incubator for 24 to 48 hours until monolayer was formed. After 90 % confluence was obtained, the growth medium was removed. BHK-21 monolayers were washed with phosphate buffer saline. A 300 mg/mL concentration of ethanolic extract was maintained in a 10 percent DMSO. Concentration of aqueous extract was also maintained at 300 mg/mL in the same solvent. *T. citrina *extracts were diluted by tenfold dilution and poured in consecutive wells of 96 well plates. Each dilution of *T. citrina* extract was evaluated for cellular toxicity in triplicate wells containing 200 µL extract which was mixed with DMEM. Cell culture medium was removed after 48 hours. Fresh DMEM (100 µL) medium and (0.25 %) MTT dye (20 µL) was added. 96 well plates were once again incubated at 37 °C in a carbon dioxide incubator for 3 hrs. Maintenance medium was removed and DMSO (100 µL) was added 3 hrs post-incubation to dissolve any formazan crystals (Akhtar et al., 2016[5]). Purple color formed due to viable cells was recorded with an ELISA reader at 570 nm. Cell survival and IC_50_ were measured. Cell survival percentage was calculated by following formula:


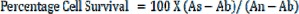


where As is the absorbance of the sample, An is the absorbance of negative control and Ab is the absorbance of blank.

IC_50_ was calculated by plotting log concentration of *T. citrina* extract on X-axis and percentage cell survival was plotted on Y-axis.

### Statistical analysis

The revertant colonies exhibited by Salmonella tester strains against different dilutions of *T. citrina* were expressed as mean and standard deviation. These were further analyzed by two way ANOVA. Cell survival percentage of BHK-21 at different concentrations of *T. citrina* extracts were evaluated by regression analysis and one way ANOVA. The data were analyzed by SPSS® and MS Office, 2013.

## Results

### Preliminary phytochemical analysis of T. citrina fruit extracts

The percentage yield of aqueous and ethanolic extracts of *T. citrina* was found to be 32.8 and 39.6 % respectively. 

The results of preliminary phytochemical screening of *T. citrina* fruit extracts are summarized in the Table 1(Tab. 1).

### Evaluation of genotoxicity

Ethanolic and aqueous extracts of *T.* *citrina* fruits were evaluated for their mutagenicity through the Ames test in Salmonella TA 100 and 102 strains. It was revealed that both the extracts exhibited strong mutagenic potential in both tester strains. Number of revertant colonies increased with an increase in the concentration of both extracts. Ethanolic extract exhibited a higher number of revertant colonies of Salmonella TA 100 than TA 102 whereas the aqueous extract of *T.* *citrina* indicated higher number of revertant colonies of Salmonella TA 102 as compared to TA 100. Table 2(Tab. 2) shows the number of revertant colonies of both tester strains upon exposure to aqueous and ethanolic extracts of *T. citrina*.

Ethanolic and aqueous extracts of *T.* *citrina* fruits were evaluated for their mutagenicity in the presence of the liver enzyme activation system. It was revealed that both the extracts exhibited strong mutagenic potential in both tester strains. Number of revertant colonies increased with an increase in the concentration of both extracts. Ethanolic extract exhibited a higher number of revertant colonies of Salmonella TA 100 than TA 102 whereas aqueous extract of *T.* *citrina* indicated higher number of revertant colonies of Salmonella TA 102 as compared to TA 100. Ethanolic extract showed no revertant colony of TA 100 whereas the aqueous extract showed no revertant colony of TA 100 at highest tested concentrations. Table 3(Tab. 3) shows the number of revertant colonies of both tester strains upon exposure to aqueous and ethanolic extracts of *T. citrina* in the presence of enzyme activation system.

Mutagenicity index of *T. citrina* fruit extracts was calculated by dividing the revertant colonies induced by them with the number of revertant colonies of negative control. It was found that even the 15 µg/ml concentration of ethanolic extract was significantly mutagenic in TA 100 tester strain, however a concentration of ethanolic extract as high as 1.5 mg/ml was found to be significantly mutagenic. Aqueous extract of *T. citrina* was safer as compared to ethanolic extract considering TA 100 tester strain. It was also revealed that aqueous extract was extremely mutagenic with Salmonella TA 102 strain even at 15 µg/ml concentration. Mutagenicity index of *T. citrina* fruit extracts are depicted in the Table 4(Tab. 4).

It was also revealed that *T. citrina* fruit extracts exhibited high mutagenic potential in the presence of the enzyme activation system. High concentrations tested against TA 100 and TA 102 showed cytotoxic effects in some cases. Both the extracts showed significant mutagenicity even the 15 µg/ml except aqueous extract which showed significant mutagenicity at 150 µg/ml concentration. Mutagenicity index of *T. citrina* fruit extracts in the presence of the enzyme activation system is depicted in the Table 5(Tab. 5).

### Cytotoxicity assay

**Figure 1:**
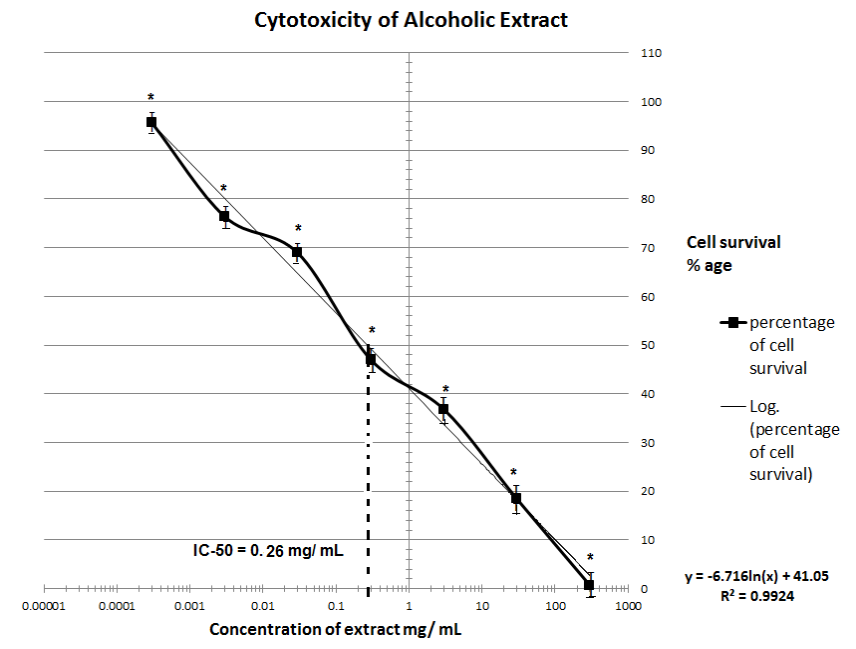
Cytotoxicity assay of ethanolic extracts of *Terminalia citrina* fruits

**Figure 2:**
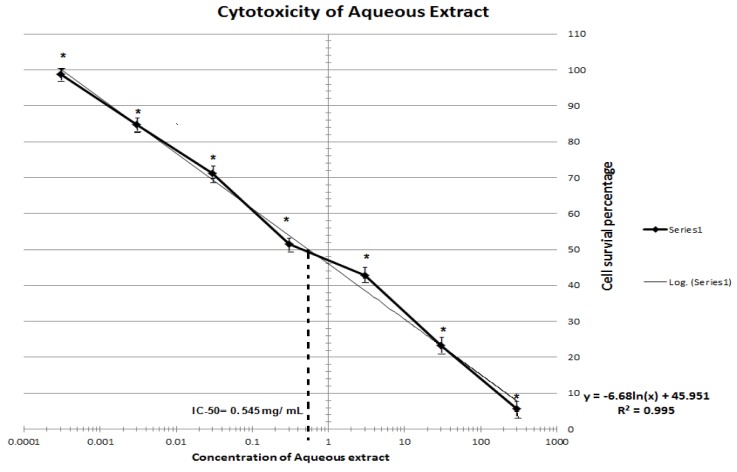
Cytotoxicity assay of aqueous extracts of *Terminalia citrina* fruits

MTT assay of *T.* *citrina* fruit extracts showed dose-dependent cytotoxic effect. Cell survival percentage increased significantly with an increase in dilution of both the extracts. Cell survival of fifty percent was exhibited by 260 and 545 µg/ml of ethanolic and aqueous extracts respectively. Thus ethanolic extract was found to retain more anti-proliferative activity than aqueous extract. MTT assay depicting the IC_50_ of ethanolic and aqueous extracts is depicted in Figures 1(Fig. 1) and 2(Fig. 2).

Statistical analysis for evaluating genotoxicity of *T. citrina* indicated that the revertant colonies shown by most of the concentrations of both the ethanolic and aqueous extracts were statistically significant at P ≤ 0.05 and mutagenicity increased with an increase in concentration of extract. Cell viability also decreased with an increase in the concentration of both extracts.

## Discussion

A huge number of studies have reported different extracts and their isolated phytochemicals for treatment of various ailments (Abad et al., 2000[1]). However, lack of widespread safety evidences put a large population at undue risk. In the present study, ethanolic and aqueous extracts of *T. citrina* fruits were subjected to genotoxic and cytotoxic evaluation so as to provide safety evidences for its ethnopharmacological use.

Genotoxicity testing revealed the strong mutagenic potential of *T. citrina* in bacterial reverse mutation system. Ethanolic extract exhibited no bacterial colonies when tester strain was TA-100 which may be attributed to its strong cytotoxic effect on this tester strain at 150 mg/mL per plate. High mutagenicity index of ethanolic and aqueous extracts in TA-100 suggested that *T. citrina* is linked to base pair mutations in bacteria. Strong genotoxic effects in TA-102 showed that transition mutations had been induced by *T. citrina* extracts. Transition mutations in TA-102 can be linked to strong oxidizing agents present in this plant which resulted in oxidative stress related single nucleotide mutations which can culminate in single nucleotide polymorphism (SNP) (Grey and Adlercreutz, 2003[14]). Aqueous and ethanolic extracts of *T. citrina* exhibited very high mutagenic potential. At 150 mg/mL concentration, aqueous extract of *T. citrina* exhibited cytotoxicity in Salmonella TA 100 strain which was evident from the absence of any revertant colonies. However, it was found that *T. citrina* was highly mutagenic at a concentration of 1.5 mg/mL which was in contrast to another study which reported that other species of this genus were not mutagenic even at more than 80 mg/0.1 mL (Sangthong et al., 2014[23]). Antioxidant mechanisms are inherent in human body and play a vital role in biological functions such as prevention of carcinogenesis, mutagenicity and aging (Fiedor and Burda, 2014[12]). Vital role for reactive oxygen species (ROS) and other oxidants in causing various hereditary defects and carcinogenesis have been established. Avoidance of strong oxidizing agents and use of antioxidants is becoming prerequisite for prevention and treatment of diseases, and preservation of human health (Del Rio et al., 2013[11]). 

Although no *in vitro* cytotoxicity of *T.* *citrina* crude extract was carried out previously however, presence of several compounds such as Punicalagin and chebulagic acid isolated from *Terminalia* species exhibited some cytotoxic activity in some human cancer cell lines (Barrajón-Catalán et al., 2010[8]). Punicalagin also induced cytotoxicity in human glioma cells via autophagic cell death and inhibited proliferation and induced apoptosis in human colon cancer cells (Wang et al., 2013[29]). It was found that *T. citrina* aqueous and ethanolic extract was cytotoxic to BHK-21 cell line with IC_50_ of 0.454 and 0.26 mg/mL which were less than the IC_50_ exhibited by *T. chebula*, *bellerica* and *rafflesia* in cancerous cell lines (Sangthong et al., 2014[23]). MTT assay unfolded potential cytotoxic and anti-proliferative activity of *T. citrina* fruit extracts. Initial screening showing cytotoxicity of the transformed cell line may depict possible anti-proliferative and anticancer activities associated with ethanolic and aqueous extracts of *T. citrina*. This activity was similar to *T. chebula,* a plant from same genera which exhibited *in vitro* anti-proliferative and anticancer activity (Gavamukulya et al., 2014[13]; Saleem et al., 2002[22]).

## Conclusion

This study concludes that *T. citrina* fruit extracts exhibit significant genotoxicity and cytotoxicity. Genotoxic and cytotoxic effects exacerbated with an increase in the concentration of extract. Genotoxicity subdued in some cases when an enzyme activation system was applied. It can be inferred from the study that *T. citrina* fruits may not be safe in human and we suggest further investigation on the safety of *T. citrina *in rodents to assess any pathological and physiological alterations associated with its use.

## Conflict of interest

Authors declare that they have no conflict of interest.

## Figures and Tables

**Table 1:**
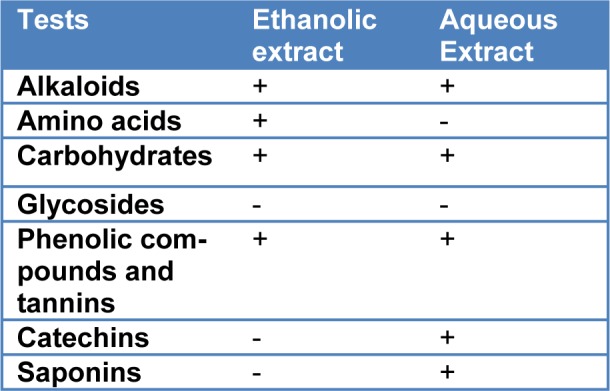
Qualitative preliminary screening of *Terminalia citrina*

**Table 2:**
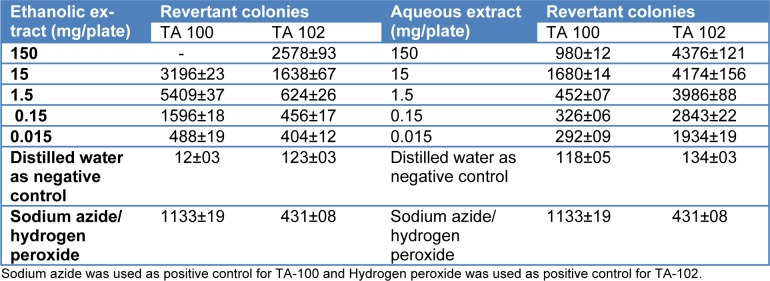
Revertant colonies of Ames tester strains on exposure to different concentrations of aqueous and ethanolic extracts of *Terminalia citrina *

**Table 3:**
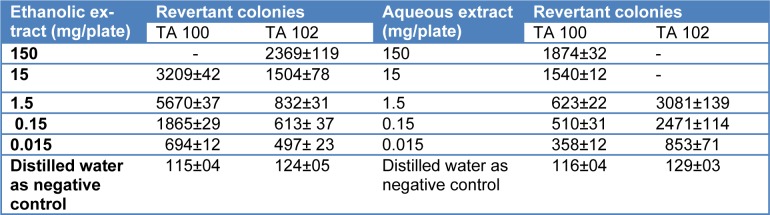
Revertant colonies of Ames tester strains on exposure to different concentrations of aqueous and ethanolic extracts of *Terminalia citrina* in the presence of enzyme activation system

**Table 4:**
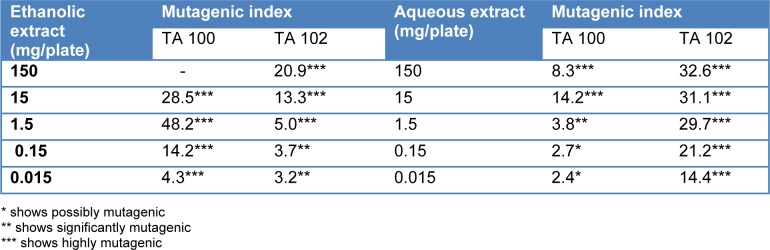
Muatagenicity index of *Terminalia citrina* on exposure to different concentrations of aqueous and ethanolic extracts

**Table 5:**
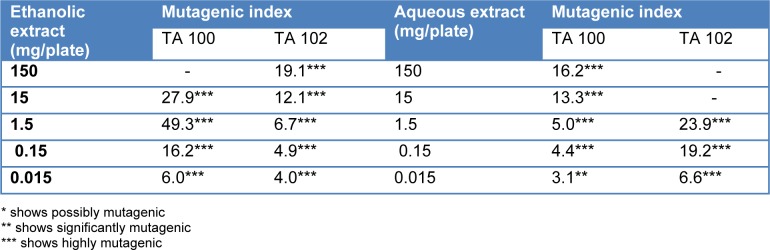
Muatagenicity index of *Terminalia citrina* on exposure to different concentrations of aqueous and ethanolic extracts in the presence of enzyme activation system
